# Conditional knockout of leptin receptor in neural stem cells leads to obesity in mice and affects neuronal differentiation in the hypothalamus early after birth

**DOI:** 10.1186/s13041-020-00647-9

**Published:** 2020-08-03

**Authors:** Zhonggan Ren, Yitong Liu, Wentong Hong, Xinjie Pan, Pifang Gong, Qiong Liu, Guomin Zhou, Song Qin

**Affiliations:** 1 Department of Anatomy, Histology and Embryology, School of Basic Medical Sciences, Fudan University, Shanghai, 200032 China; 2 Department of Anatomy, Histology and Embryology, School of Basic Medical Sciences, Fudan University, Shanghai, China;, Key Laboratory of Medical Imaging Computing and Computer Assisted Intervention of Shanghai, Shanghai, 200032 China

**Keywords:** Hypothalamus, Leptin receptor, Arcuate nucleus, Neural stem cells, Neuronal differentiation

## Abstract

Leptin, secreted by peripheral adipocytes, binds the leptin receptor (Lepr) in the hypothalamus, thereby contributing to the regulation of satiety and body weight. Lepr is expressed in the embryonic brain as early as embryonic day 12.5. However, the function of Lepr in neural precursor cells in the brain has not been resolved. To address this issue, we crossed the *Lepr*^*flox/flox*^ mice with each of *Shh-Cre* mice (Shh, sonic hedgehog) and *Nestin (Nes)-Cre* mice. We found that deletion of Lepr specifically in nestin-expressing cells led to extreme obesity, but the conditional null of Lepr in Shh-expressing cells had no obvious phenotype. Moreover, the level of leptin-activated pSTAT3 decreased in the anterior and central subregions of the arcuate hypothalamus of *Shh-Cre; Lepr*^*flox/flox*^ mice compared with the controls. By contrast, in *Nes-Cre; Lepr*^*flox/flox*^ mice, the level of leptin-activated pSTAT3 decreased in all subregions including the anterior, central, and posterior arcuate hypothalamus as well as the dorsomedial, ventromedial, and median eminence of the hypothalamus, revealing that the extensive lack of Lepr in the differentiated neurons of the hypothalamus in the conditional null mice. Notably, conditional deletion of Lepr in nestin-expressing cells enhanced the differentiation of neural precursor cells into neurons and oligodendroglia but inhibited differentiation into astrocytes early in postnatal development of hypothalamus. Our results suggest that Lepr expression in neural precursor cells is essential for maintaining normal body weight as well as the differentiation of neural precursor cells to the neural/glial fate in the hypothalamus shortly after birth.

## Introduction

Leptin, an adipose-derived hormone, is a critical regulator of diverse metabolic processes including satiety, energy expenditure, and glucose homeostasis [1, 2]. In the brain, leptin binds and activates the long isoform of leptin receptor (Lepr), which is mainly expressed in tuberal region of hypothalamus including the arcuate hypothalamus (ARH), dorsomedial hypothalamus (DMH), the ventromedial hypothalamus (VMH), median eminence (ME) [3, 4]. Notably, leptin targets orexigenic agouti-related protein (AgRP) and anorexigenic pro-opiomelanocortin (POMC) neurons of the ARH to decrease feeding and increase energy expenditure [5–7].

Like Leptin, Sonic hedgehog (Shh) ligand and its signaling pathway regulate aspects of growth and metabolism that are relevant to hypothalamic patterning [8–11]. Conditional deficiency of *Shh* in mouse diencephalon leads to a large reduction in the numbers of AgRP and POMC neurons [8]. After embryonic day 9.5, Shh-expressing hypothalamic progenitors give rise to neurons and astrocytes of the entire tuberal region and in particular the ventromedial nucleus [12]. Lepr is expressed in the ventricular zone of the telencephalon and mesencephalon at embryonic day 12.5 [13] as well as in the ARH during early postnatal development in rodents [14]. However, the function of Lepr in neural precursor cells (NPCs), especially the Shh-expressing NPCs in the brain, has not been determined.

In the present study, we investigate the role of Lepr in NPCs by crossing *Lepr*^*flox/flox*^ mice with each of *Shh-Cre* mice and *Nestin-Cre* mice. The *Nestin-Cre; Lepr*^*flox/flox*^ (Nes-cKO) mice become strongly obese, with markedly increased body weight from postnatal week 5, whereas the *Shh-Cre; Lepr*^*flox/flox*^ (Shh-cKO) mice have no obvious phenotype. We divide the hypothalamic tuberal region into six subregions: anterior region of ARH (ARH-A), central region of ARH (ARH-C), posterior region of ARH (ARH-P), DMH, VMH and ME. The extent of phosphorylation (p) of the transcription factor STAT3 (pSTAT3) after stimulation of cells with leptin is reduced in the ARH-A and ARH-C subregions of Shh-cKO mice, whereas the number of pSTAT3-expressing neurons is significantly reduced in all six subregions of Nes-cKO mice compared with control mice. Furthermore, we show that conditional knockout of Lepr in NPCs alter their differentiation towards neuronal/glia fate in the hypothalamus early during postnatal development. Our findings suggest that Lepr expressed in NPCs is essential for maintaining normal body weight and balancing the neural/glial fate differentiation of NPCs in early postnatal development.

## Results

### *Nes-Cre;Lepr*^*f/f*^ mice become obese by postnatal day 35 (P35) but no obvious obesity phenotype is observed for *Shh-Cre;Lepr*^*f/f*^ mice

**Fig. 1:**
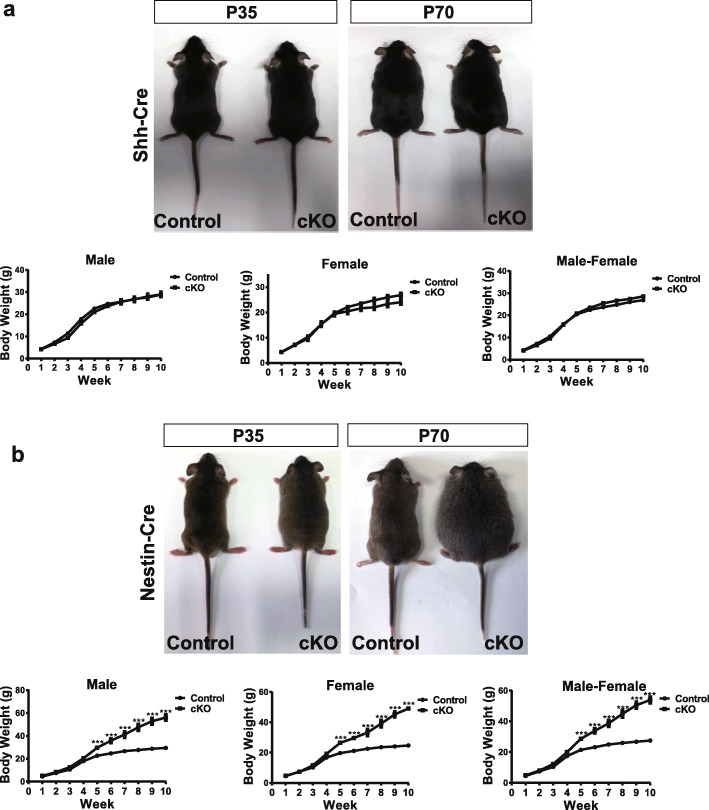
Specific depletion of Lepr in nestin-expressing progenitor cells leads to obesity. **a** No obvious phenotype of the *Shh-Cre;Lepr*^*flox/flox*^*(*Shh-cKO) mice compared with control littermates examined at P35 or P70, as indicated (upper panel). No difference in body weight of the Shh-cKO mice compared with control littermates examined during the first 10 weeks after birth (lower panel). **b***Nes-Cre;Lepr*^*f/f*^ (Nes-cKO) mice display severe obesity (upper panel) at P35 or P70 and a significant increase of body weight from week 5 compared with control littermates (lower panel). (*n* ≥ 10 mice per group)

Shh is required for the normal function of orexinergic and anorexinergic cells in the hypothalamus as well as for maintaining the proper size of the lateral hypothalamus [8, 15]. To characterize the pattern of *Shh* expression in the hypothalamus, we first crossed *Shh-Cre* mice with the tdTomato reporter line *Ai14* transgenic (tg) mice for cell lineage mapping. Shh was expressed abundantly in the hypothalamus, especially in the tuberal region (Supplementary Fig. S1). To assess Lepr function in Shh signaling, we generated a *Lepr* conditional knockout (cKO) mouse line by crossing *Shh-Cre* with *Lepr*^*flox/flox*^ tg mice. We assessed obesity visually and monitored body weight weekly for the first 10 weeks after birth. These *Shh-cKO* mice had no obvious obesity phenotype compared with wild-type or heterozygous control littermates (Fig. 1a). Moreover, body weight of the Shh-cKO mice showed no difference with the control group (Fig. 1a), suggesting that Lepr is not required for normal neurogenesis in cells that express Shh.

To understand the role of Lepr in NPCs, we generated another *Lepr* cKO line by crossing *Nes-Cre* with *Lepr*^*flox/flox*^ tg mice. Unexpectedly, Nes-cKO mice were severely obese, with markedly increased body weight from week 5 after birth (Fig. 1b). By dividing Nes-cKO mice into male and female groups, mice in each group showed similar obesity phenotypes (Fig. 1b). These results revealed that Lepr expressed in NPCs is essential for maintaining normal body weight, and depletion of Lepr in NPCs leads to obesity.

### Altered expression of Leptin-activated pSTAT3 in the tuberal region of hypothalamus of *Shh-Cre; Lepr*^*flox/flox*^ mice

**Fig. 2:**
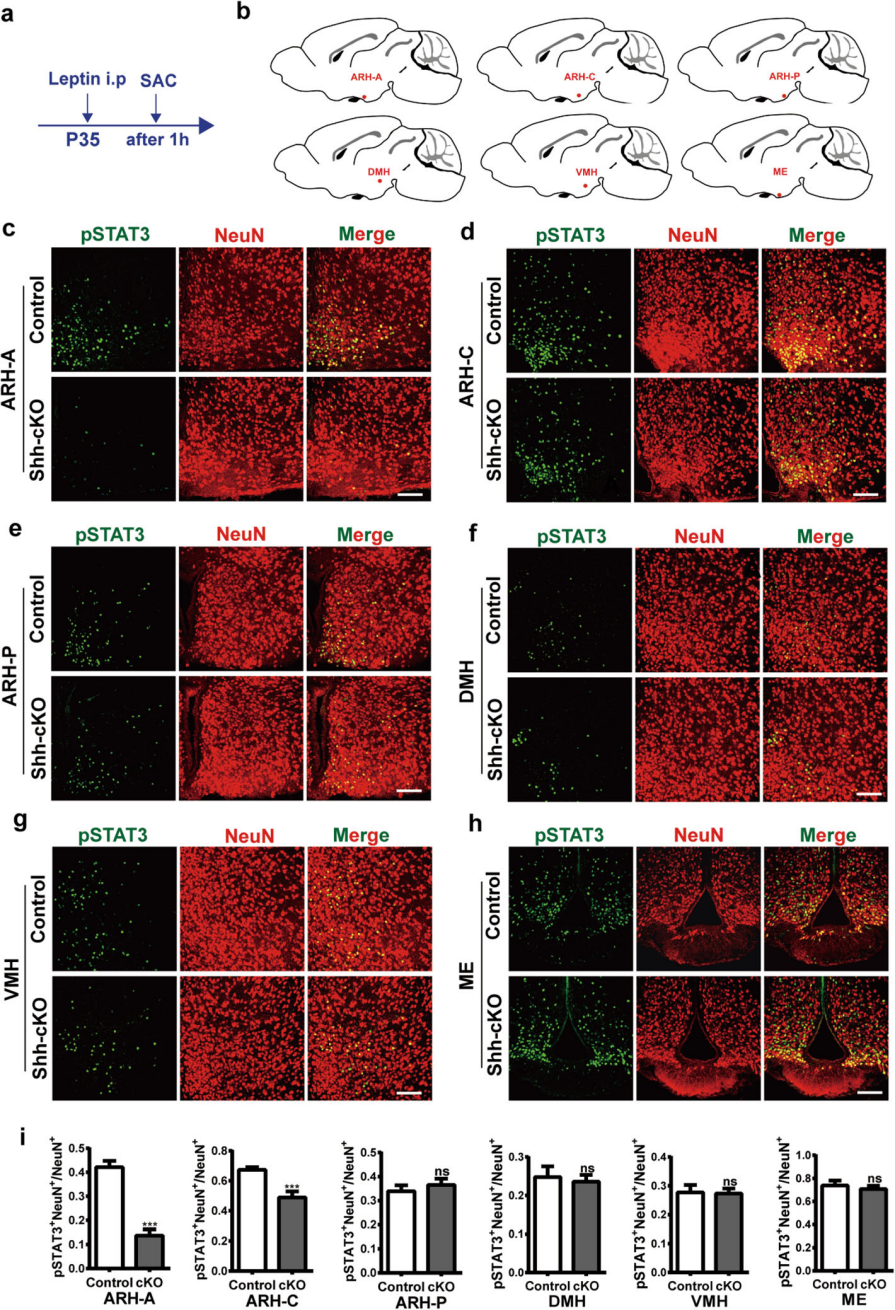
The level of activated pSTAT3 stimulated by leptin decreased in the ARH-A and ARH-C of Shh-cKO mice compared with control littermates. **a** Experimental scheme. Shh-cKO mice or control littermates at P35 received a single i.p. injection of leptin and were sacrificed after 1 h. **b** Schematic diagrams depicting the indicated brain regions for analysis. **c-h** Representative images showing leptin-activated pSTAT3 and NeuN expression in the indicated subregions of the hypothalamus of Shh-cKO mice and control littermates. Scale bars, 100 μm. **I** Quantification of the ratio of pSTAT3 + NeuN+ cells to total NeuN+ cells (*n* = 4 mice for each genotype). ***p < 0.001; Student’s two-tailed, unpaired *t*-test. Error bars indicate s.e.m.

Treatment of Lepr-expressing hypothalamus cells with leptin in vivo activates leptin-LepR signal transduction, which induces the phosphorylation of a tyrosine in STAT3 (pSTAT3) [16, 17]. Therefore, the induction of pSTAT3 represents a convenient histochemical marker for lepr activation [7]. To examine the effects of exogenous leptin in *Shh-Cre; Lepr*^*flox/flox*^ tg mice, recombinant Leptin (3 mg/kg) was injected intraperitoneally at P35 (Fig. 2a and b). At 1 h post-injection, each mouse brain was excised, and brain slices were subjected to immunostaining for pSTAT3 and neuronal-nuclei protein (NeuN). Most of the pSTAT3+ cells were NeuN+ neurons in the hypothalamus (Fig. 2c-h). The ratio of pSTAT3+ cells displaying co-localization with NeuN staining to total NeuN+ cells was remarkably reduced in the ARH-A and ARH-C of Shh-cKO mice compared with wild-type controls (Fig. 2c, d and i). However, the level of pSTAT3 did not change significantly in other subregions such as ARH-P, DMH, VMH, or ME in Shh-cKO mice compared with controls (Fig. 2e-i).

### Altered expression of Leptin-activated pSTAT3 in the tuberal region of hypothalamus of *Nes-Cre; Lepr*^*flox/flox*^ mice

**Fig. 3:**
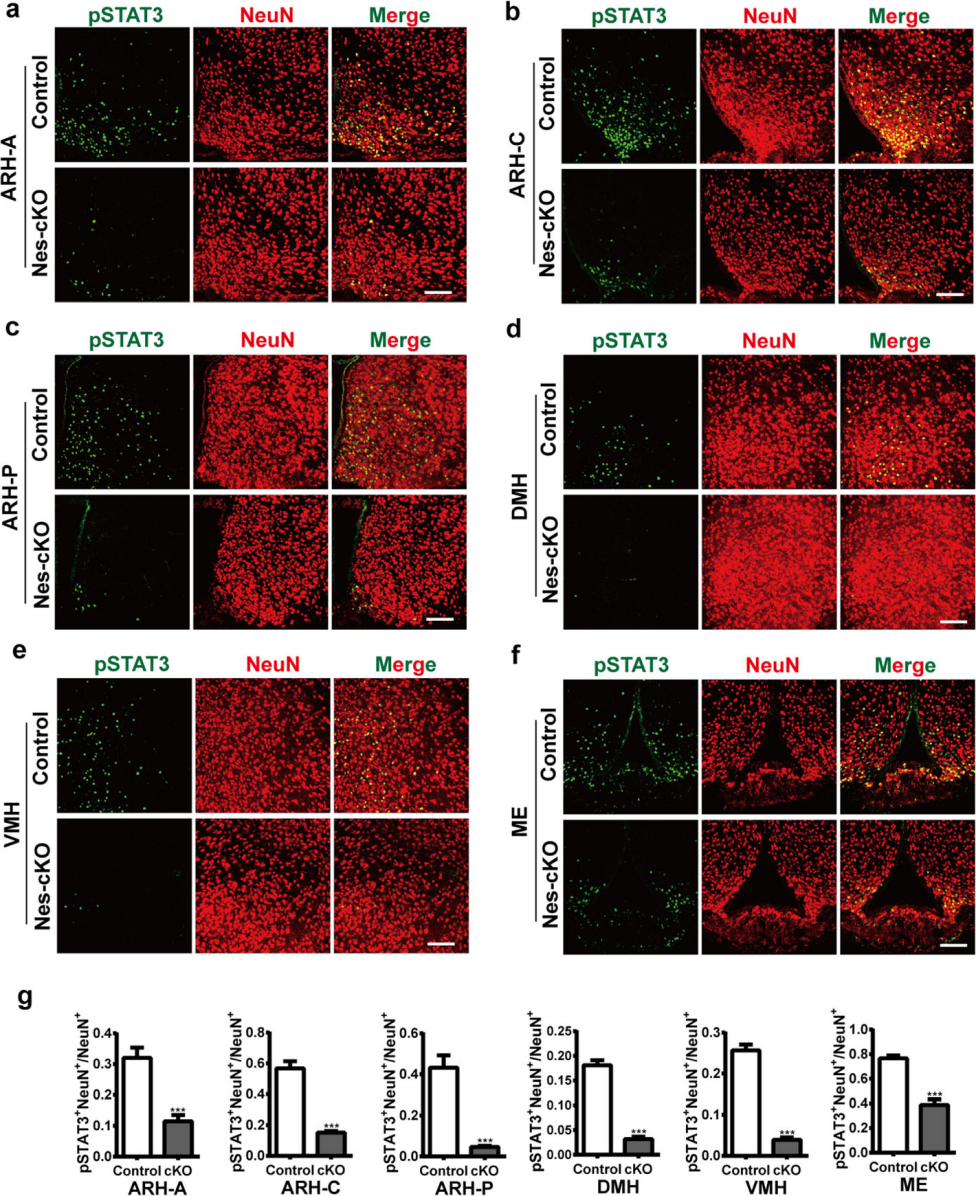
The level of activated pSTAT3 stimulated by leptin markedly decreased in all subregions of the hypothalamus of Nes-cKO mice compared with control littermates. **a-f** Representative images showing leptin-activated pSTAT3 and NeuN expression in the indicated subregions of the hypothalamus of Nes-cKO mice and control littermates. Scale bars, 100 μm. **g** Quantification of the ratio of pSTAT3 + NeuN+ cells to total NeuN+ cells (*n* = 4 mice for each genotype). ***p < 0.001; Student’s two-tailed, unpaired *t*-test. Error bars indicate s.e.m.

To assess the general role of Lepr in NPCs, we examined the level of pSTAT3 at 1 h post-stimulation with leptin in *Nes-Cre; Lepr*^*flox/flox*^ tg mice. Similar to observations with *Shh-Cre; Lepr*^*flox/flox*^ tg mice, most of the pSTAT3+ cells were also NeuN+ in the hypothalamus (Fig. 3a-f). Notably, we found that the ratio of NeuN+pSTAT3+ cells to total NeuN+ cells was remarkably reduced in each subregion: ARH-A, ARH-C, ARH-P, DMH, VMH, or ME in the hypothalamus of Nes-cKO mice compared with controls (Fig. 3a-g). These results suggest that conditional Lepr depletion in NPCs strongly affects leptin-induced STAT3 signaling in vivo.

Because STAT3 undergoes tyrosine phosphorylation under physiological conditions, we assessed pSTAT3 level in Nes-cKO mice that were not treated with leptin. At 1 h after treatment of these mice with 0.9% saline, scattered pSTAT3+ cells were observed in the ARH-C and ME (Fig. S2), similar to observations with leptin-treated Nes-cKO mice.

### Neuronal differentiation of NPCs is not altered in the hypothalamus during adulthood of *Nes-Cre; Lepr*^*flox/flox*^ mice

**Fig. 4:**
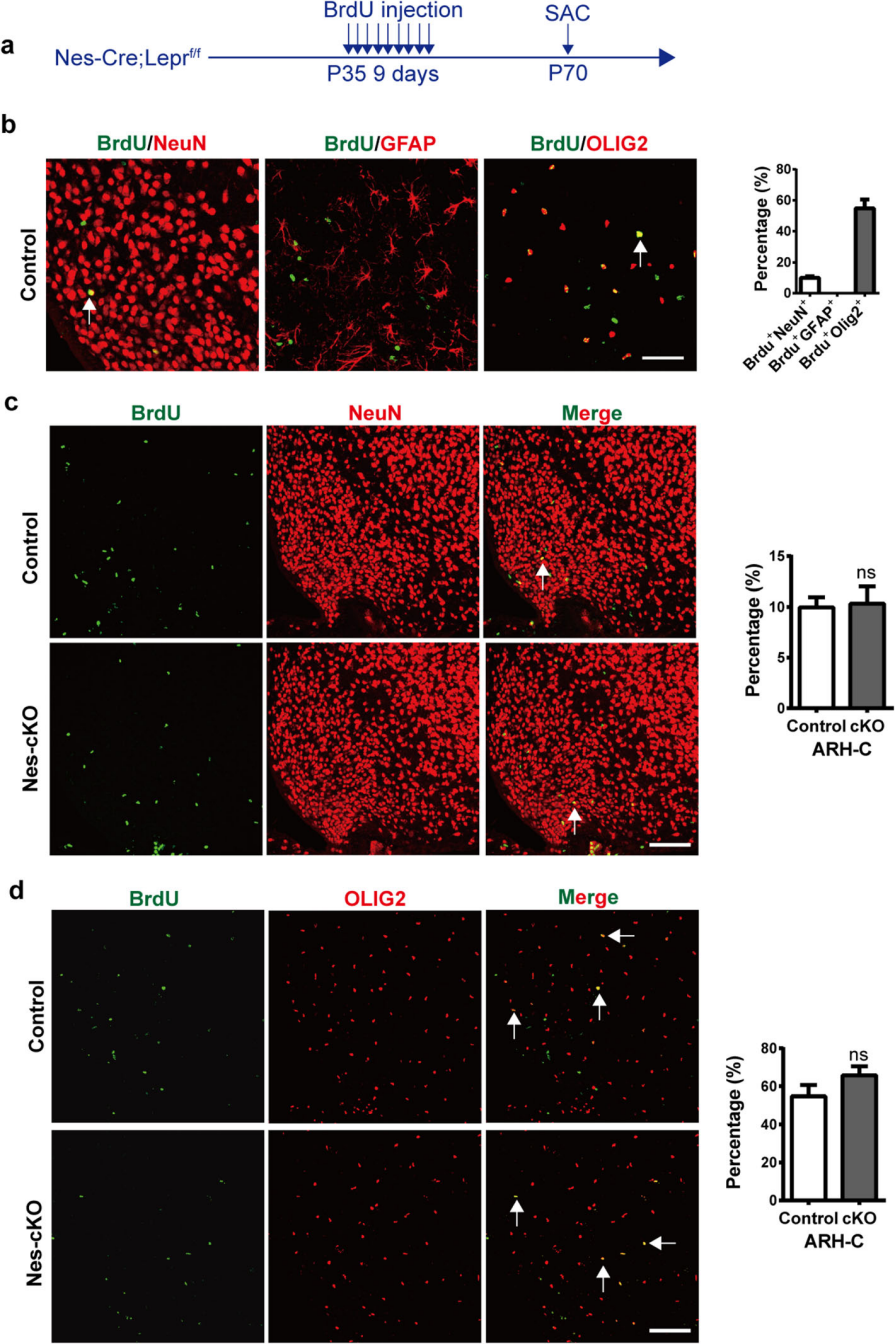
Depletion of Lepr in nestin-expressing cells does not affect the fate of NPCs in the hypothalamus of adult mice. **a** Experimental scheme. Nes-cKO mice or control littermates received BrdU twice daily between P35 and P43 and were sacrificed at P70. **b** Representative images showing that BrdU-expressing cells colocalize with NeuN, GFAP, and OLIG2. Shown to the right is the quantification of the percentage of BrdU+ cells displaying co-localization with each cell type. Scale bar, 50 μm. **c** No significant change in the ratio of BrdU+NeuN+ cells to total BrdU+ cells in Nes-cKO mice compared with control littermates. Scale bar, 100 μm. **d** No significant change in the ratio of the BrdU+OLIG2+ cells to total BrdU+ cells in the Nes-cKO mice compared with control littermates (*n* = 4 mice per group). Scale bar, 100 μm. Student’s two-tailed, unpaired *t*-test. Error bars indicate s.e.m.

Previous studies have analyzed the incorporation of the mitotic marker bromodeoxyuridine (BrdU) in NPCs and found that neurogenesis occurs in the hypothalamus during the postnatal period [18, 19]. To examine whether Lepr is involved in neurogenesis in the adult hypothalamus, we first injected P60 C57BL/6 wild-type mice with BrdU (50 mg/kg, i.p.) twice daily for five consecutive days. The proliferation of progenitor cells mostly occurred in the ARH-C of the hypothalamus (Fig. S3). To assess the differentiation status of the BrdU+ cells and examine whether differentiation was altered in the hypothalamus of the adult *Nes-Cre; Lepr*^*flox/flox*^ mice, the mice received BrdU (50 mg/kg, i.p.) twice daily between P35 and P43 and then were sacrificed at P70 (Fig. 4a). Hypothalamic sections from the mice were analyzed using immunofluorescence microscopy after incubation with an antibody against BrdU and/or other markers such as NeuN, GFAP (glial fibrillary acidic protein), or OLIG2 (Oligodendrocyte transcription factor 2). Approximately 10% of the BrdU+ cells were NeuN+ neurons, 55% were OLIG2+ oligodendrocytes, and none were positive for the astrocyte marker GFAP (Fig. 4b). Further analysis of samples from the *Nes-Cre; Lepr*^*flox/flox*^ mice revealed that the proportion of NeuN+BrdU+ among the BrdU+ cells did not change significantly compared with control mice (Fig. 4c). Moreover, the ratio of OLIG2 + BrdU+ cells to total BrdU+ cells did not differ between the Nes-cKO mice and control mice (Fig. 4d*).* These results suggest that conditional depletion of Lepr in NPCs had no effect on NPC lineage choice in the hypothalamus of adult mice.

### Conditional depletion of Lepr in NPCs influences the differentiation of NPCs in the hypothalamus early in postnatal development

**Fig. 5:**
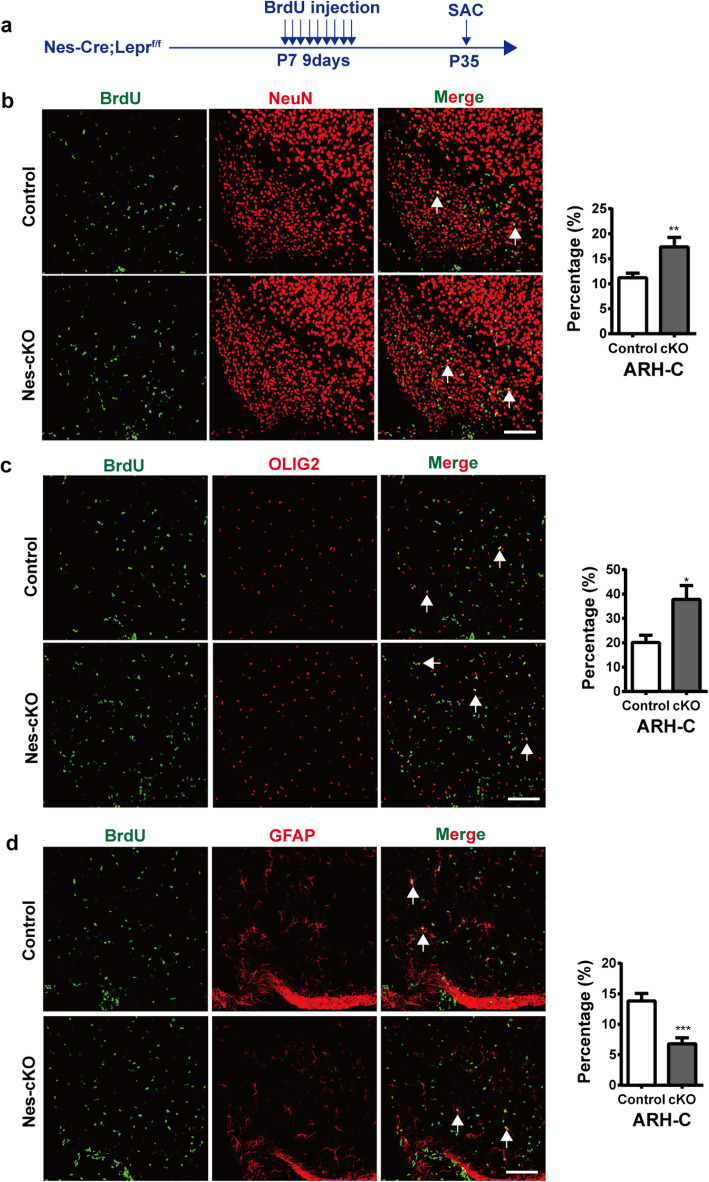
Depletion of Lepr in nestin-expressing cells enhances the differentiation of these cells towards the neuronal and oligodendroglia fates but inhibits the astrocyte fate early in postnatal development **a** Experimental scheme. Nes-cKO mice or control littermates received BrdU twice daily between P7 and P15 and were sacrificed at P35. **b-d** Representative images showing BrdU-expressing cells colocalizing with NeuN (**b**), OLIG2 (**c**) or GFAP (**d**) by immunostaining of samples from control or Nes-cKO mice. Shown to the right is the quantification of the percentage of the BrdU+ cells displaying co-localization with NeuN, OLIG2 or GFAP (*n* = 4 mice per group), Scale bars, 100 μm. *p < 0.05, **p < 0.01, ***p < 0.001; Student’s two-tailed, unpaired *t*-test. Error bars indicate s.e.m.

During mouse development, the level of circulating leptin increases dramatically during postnatal week 2 independently of fat mass, and leptin level then decreases after weaning [20]. Interestingly, i.p. administration of leptin stimulates the proliferation of astrocytes in the hypothalamus, whereas depletion of Lepr in GFAP-expressing cells reduces astrocyte proliferation during early postnatal period [21]. To examine whether the deletion of Lepr in NPCs could influence NPCs differentiation in the developing hypothalamus, *Nes-Cre; Lepr*^*flox/flox*^ mice were injected (i.p.) with BrdU (50 mg/kg body weight) twice daily from P7 to P15 and then sacrificed at P35 (Fig. 5a). Immunofluorescence staining for BrdU and/or other markers such as NeuN, GFAP, and OLIG2 revealed that both proportions of BrdU+NeuN+ and BrdU+OLIG2+ cells among total BrdU+ cells were significantly greater than that of control mice (Fig. 5b and c), whereas the relative proportion of BrdU+GFAP+ was much smaller in the *Nes-Cre; Lepr*^*flox/flox*^ mice compared with controls (Fig. 5d). These results suggested the conditional depletion of Lepr in NPCs enhanced the differentiation of NPCs toward neuronal and oligodendroglial fates but inhibited differentiation towards the astrocyte fate early in postnatal development.

### Fate differentiation of NPCs is not altered in the dentate gyrus (DG) early in postnatal development of *Nes-Cre; Lepr*^*flox/flox*^ mice

**Fig. 6:**
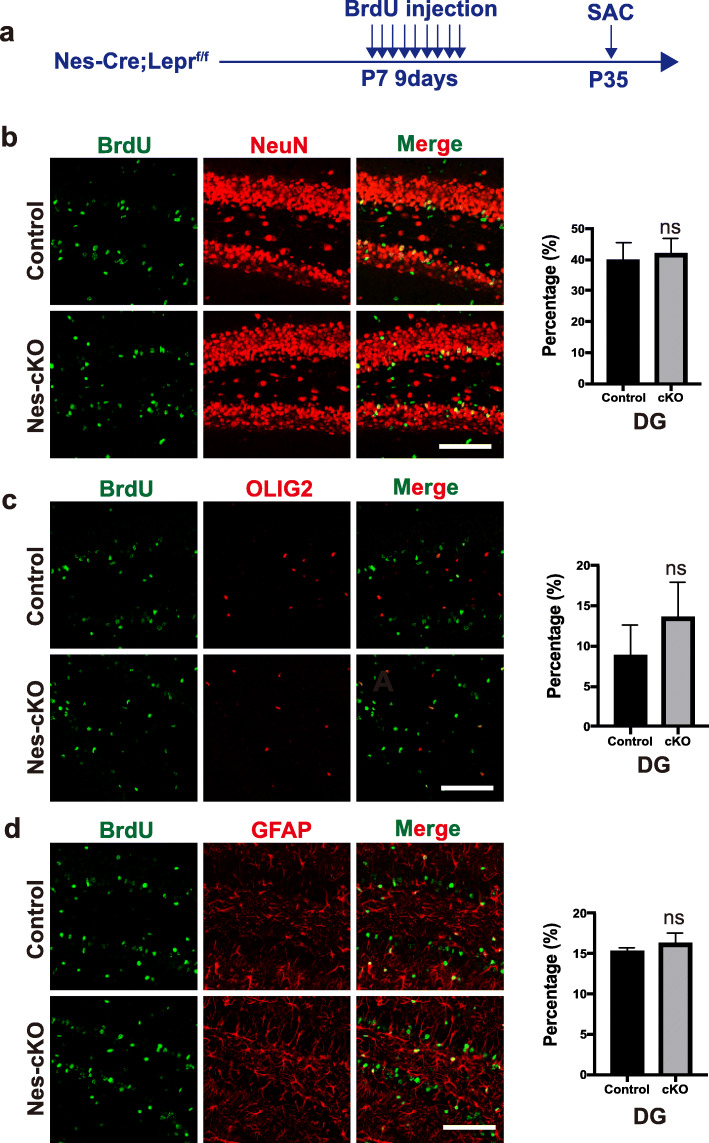
Depletion of Lepr in nestin-expressing cells does not affect the fate of NPCs in the dentate gyrus during early postnatal development. **a** Experimental scheme. Nes-cKO mice or control littermates received BrdU twice daily between P7 and P15 and were sacrificed at P35. **b-d** Representative images showing BrdU-expressing cells colocalizing with NeuN (**b**), OLIG2 (**c**) or GFAP (**d**) in the dentate gurus by immunostaining of samples from control or Nes-cKO mice. Shown to the right is the quantification of the percentage of the BrdU+ cells displaying co-localization with NeuN, OLIG2, or GFAP (*n* = 4 mice per group). DG, dentate gurus. Scale bars, 100 μm. ns, no significant; Student’s two-tailed, unpaired *t*-test. Error bars indicate s.e.m.

Neurogenesis arising from neural stem cells in the subgranular zone (SGZ) of the DG occurs continuously in the postnatal period [22]. To examine whether the conditional depletion of Lepr in NPCs has a general effect in different brain regions, we analyzed the hippocampal sections from Nestin-cKO mice that was injected with BrdU from P7 to P15 and then sacrificed at P35 (Fig. 6a). Fluorescence immunostaining for BrdU and NeuN in the DG of *Nes-Cre; Lepr*^*flox/flox*^ mice revealed the proportion of NeuN+BrdU+ among the BrdU+ cells did not change significantly compared with control mice (Fig. 6b). Moreover, there was no significant change in the proportion of BrdU+OLIG2+ or BrdU+GFAP+ cells among total BrdU+ cells compared with control mice (Fig. 6c and d). Thus, these results indicated that the conditional knockout of Lepr in NPCs has no effect on the fate differentiation in the DG during early postnatal development.

## Discussion

Congenital deficiency of leptin or Lepr owing to loss-of-function mutations in rodents and humans leads to profound metabolic dysfunctions, including hyperphagia, obesity, insulin resistance and infertility [23–25]. Shh, which is secreted by the prechordal plate, is vital for cell-type patterning the hypothalamic primordium [11, 26]. Our fate mapping of *Shh-Cre;Ai14* mice revealed that the Shh promoter can strongly drive tdTomato expression in the tuberal region of the hypothalamus. Because Shh can be expressed and produced by neurons and astrocytes [27], Lepr was not only deleted in Shh-expressing NPCs, but also in other neural cells. Our results showed that conditional Lepr depletion in Shh-expressing cells of the Shh-cKO mice exhibit no obvious phenotype, indicating that non-Shh-expressed NPCs or neural cells may be mainly involved in the metabolic process of leptin signaling regulation. Although the level of leptin-activated pSTAT3 in the ARH-A and ARH-C was reduced in Shh-cKO mice, further experiments are needed to determine the physiological effects in vivo of leptin-STAT3 signaling in the ARH-A and ARH-C.

In contrast to the Shh-cKO mice, in Nes-cKO mice, the level of leptin-activated pSTAT3 was decreased in all hypothalamus subregions including the ARH-A, ARH-C, ARH-P, DMH, VMH and ME, revealing that the broad lack of Lepr in the differentiated neurons of the hypothalamus. Accordingly, the Nes-cKO mice became extremely obese, with markedly increased body weight. It has been reported that the specific subset of orexigenic or anorexigenic neurons can be regulated by multiple transcription factors. For instance, the transcription factor Bsx is essential for normal expression of neuropeptide Y and agouti-related protein [28], and Bsx-deficient mice exhibit reduced food intake [29], Ngn3+ progenitors contribute to the production of both pro-opiomelanocortin and neuropeptide Y neuronal subtypes in the ventral hypothalamus [30], and Dbx1 specifies the neuropeptide Y subpopulation as well as agouti-related protein–expressing orexigenic neurons in the ARH [31]. It will be interesting to examine whether Lepr interacts genetically with these factors.

Previous studies used genetics-based cell-lineage analyses to identify tanycytes as NPCs in the ependymal layer of the third ventricle in the adult rodent brain [32–34]. Increasing evidence has suggested that the neurogenic niche in the mammalian hypothalamus is vital for the regulation of metabolism and body weight [35, 36]. Our results using the BrdU pulse-chase analysis suggest that the conditional null of LepR in NPCs has no effect on NPC lineage choice in the hypothalamus of adult mice. Thus, the obesity phenotype observed for Nes-cKO mice cannot be attributed to any alteration of neurogenesis in the adult hypothalamus.

GFAP-expressing cells in the periventricular zone of the third ventricle also express Lepr and are responsive to leptin during the initial postnatal week [21]. Interestingly, our results reveal that conditional Lepr depletion in NPCs enhanced the differentiation of NPCs toward the neuronal and oligodendroglia fates yet inhibited the astrocyte fate early in postnatal development. This inhibition of astrocyte fate by Lepr depletion specifically in NPCs is consistent with the report that leptin potentiates astrogenesis in the developing hypothalamus [21]. It is worth noting that glial cells such as GFAP-expressing astrocytes and OLIG2-expressing oligodendrocytes are also proliferative at early postnatal stage [37, 38]. Administration of leptin can stimulate hypothalamic astrocyte proliferation during the early postnatal period [21]. In addition to the oligodendrocyte differentiation, the depletion of Lepr in NPC might also affect the proliferation of oligodendrocytes in the postnatal period.

Leptin treatment has been shown to stimulate the proliferation of hippocampal neural stem cells in vitro and in vivo [39]. Although our result reveals that neuronal differentiation is enhanced in the early postnatal hypothalamus, the fate differentiation of NPCs in the hippocampal formation is not altered in Nes-cKO mice. Future experiments are also needed to determine whether the conditional deletion of Lepr in NPCs of Nes-cKO mice affects the self-renewal/proliferation of neural stem cells in the neurogenic regions such as the hippocampal formation or hypothalamus.

Of note, however, our result that Lepr depletion in Nestin+ cells enhanced the differentiation of those cells toward the neuronal and oligodendroglia fates is inconsistent with the report that Lepr depletion in GFAP-expressing cells did not affect the number of hypothalamic neurons early in postnatal life [21]. As nestin is regarded as a multipotent neural stem cell marker during both early development and adulthood [40], GFAP-expressing cells in the periventricular zone of the third ventricle may represent distinct progenitors of astrocytes in the hypothalamus early in the postnatal period. Our study suggests that Lepr expression in NPCs is essential for maintaining normal body weight as well as the fate commitments from NPCs in the hypothalamus shortly after birth. A recent study has revealed that the absence of leptin signaling in early life cause permanent changes in energy homeostasis, melanocortin system, reproduction and brain development [41]. Nevertheless, our findings that the lineage commitments occurred early in development may be involved in the obesity associated with pathological conditions. The future identification of this distinct cell population, for example, whether the numbers of pro-opiomelanocortin neurons and agouti-related protein neurons are altered, will help us understand how metabolism is regulated by leptin signaling under physiologic and pathologic conditions.

## Materials and methods

### Animals

All mice were maintained on a C57BL/6 genetic background. The *Lepr*^*flox/flox*^ line (B6.129P2-Leprtm1Rck/J, stock no. 008327 from the Jackson Laboratory,) was kindly provided by L. Ma (Fudan University). The Shh-Cre line [42] (Stock No. 005622 from the Jackson Laboratory) and *Nestin-Cre* line (B6.Cg-Tg (Nes-cre)1Kln/JNju, Stock No. J003771 from Model Animal Research Center of Nanjing University) were kindly provided by Z. Yang (Fudan University). All mice were housed under a 12-h light/dark cycle and had ad libitum access to food and water in a controlled animal facility. All animals were treated in accordance with protocols approved by the Animal Care and Use Committee of Shanghai Medical College of Fudan University.

### Tissue preparation

Mice were deeply anesthetized with pentobarbital sodium by intraperitoneal (i.p.) injection. Animals were perfused with 0.01 M PBS and 4% paraformaldehyde (PFA), then the brains were collected and postfixed in 4% PFA at 4 °C overnight. Brains were dehydrated in 30% sucrose in PBS at 4 °C for 3 days. After embedding in optimal cutting temperature compound (OCT, Tissue-Tek), specimens were frozen then cut with a cryostat at a thickness of 40 μm.

### Immunohistochemistry

Free-floating sections were washed with PBS and blocked with 5% Bovine Serum Albumin (BSA) in PBS. After overnight incubation with primary antibodies diluted in the blocking solution with gentle agitation at 4 °C, sections were washed and incubated for 2 h with corresponding secondary species-specific antibodies conjugated with Alexa Fluor 488, 555, or 647 (Jackson ImmunoResearch). Nuclei were counterstained with Hoechst 33342. The following primary antibodies were used: pSTAT3 (Cat. #: 9131, 1:250, Cell Signalling Technology), NeuN (Cat. #: MAB377, 1:500, Thermo Scientific), GFAP (Cat. #: G3893, 1:500, Thermo Scientific), Olig2 (Cat. #: AB9610, 1:500, Thermo Scientific), BrdU (Cat. #: OBT0030, 1:1000, Accurate Chemical).

### Leptin administration

Leptin (Peprotech) was dissolved in saline, and all leptin administration was done by intraperitoneal (i.p.) injections. Postnatal (P) 35 mice were received a single i.p. injection of Leptin (3 mg/Kg) and were sacrificed after 1 h (without food access).

### Brdu incorporation assay

For P60 mice, i.p. injection of BrdU (50 mg/kg) twice daily for 5 days, sacrifice after 2 h of final injection. For P35 mice, i.p. injection of BrdU (50 mg/kg) twice daily for 9 days, sacrifice at P70. For P7 mice, Subcutaneous injection BrdU (50 mg/kg) twice daily for 9 days, sacrifice at PD35. Prior to immunostiaining, free-floating brain sections were denatured with 2 N hydrochloric acid for 30 min at 37 °C then renatured with boric acid at room temperature for 30 min.

### Confocal microscopy and image quantification

We focus on the following brain regions, ARH-A (bregma − 1.46 mm), ARH-C, ME VMH, DMH (bregma − 2.06 mm) and ARH-P (bregma − 2.46 mm). Fluorescent Z-stack images were acquired on a Nikon A1 confocal laser microscope equipped with a 25× immersion field water immersion objective. The same laser and scanning settings were used for all images within an experiment to allow comparison across groups. The confocal images were analyzed by NIH ImageJ software. A Cell Counter software plugin in the ImageJ program was used to count cells. For all dependent measures, four slides per region of interest per mouse were analyzed from ≥3 mice per experimental condition.

### Statistical analyses

Statistical analyses and plots were performed using the GraphPad Prism 5.0 software. The data are expressed as the mean value ± s.e.m. Statistical analyses were performed using Students unpaired two-samples t-test. A level of *p* < 0.05 was considered significant.

## Supplementary information

**Additional file 1: Figure S1.** Genetic tracing of the Shh promoter driving tdTomato (tdT) reporter expression in the brain of *Shh-Cre;Ai14* mice at P35. **a** tdT-expressing cells are widely distributed in the cortex, hypothalamus, and cerebellum as seen in representative sagittal sections Scale bar, 1 mm. Higher-magnification views of tdT^+^ cells in the boxed regions are shown in a1, a2, and a3. Scale bar, 200 μm. **b** Representative images of coronal sections showing the tdT-expressing cells in the ARH-A, ARH-C, and ARH-P in the tuberal region of the hypothalamus. Scale bar, 200 μm.

**Additional file 2: Figure S2.** Endogenous expression of pSTAT3 in Nes-cKO mice. Mice at P35 received 0.9% saline and were sacrificed after 1 h. pSTAT3 immunostaining of coronal brain sections showing pSTAT3+ cells in the ARH and ME in the hypothalamus. Scale bars, 100 μm.

**Additional file 3: Figure S3.** Proliferation of cells in the ARH of adult mice. C57BL/6 wild-type mice at P60 were injected with BrdU twice daily for five consecutive days and then sacrificed. BrdU immunostaining showing the BrdU+ cells in the ARH-A, ARH-C and ARH-P. Scale bar, 100 μm.

## Data Availability

The data that support the findings of this study are available from the corresponding author upon reasonable request.
