# Supplementary material for: Conditional knockout of leptin receptor in neural stem cells leads to obesity in mice and affects neuronal differentiation in the hypothalamus early after birth
Source: Mol Brain. 2020 Aug 3;13:109. doi: 10.1186/s13041-020-00647-9 (PMC7398062; doi:10.1186/s13041-020-00647-9)
Supplement: Supplementary file 1 — Additional file 1: Figure S1. Genetic tracing of the Shh promoter driving tdTomato (tdT) reporter expression in the brain of Shh-Cre;Ai14 mice at P35. a tdT-expressing cells are widely distributed in the cortex, hypothalamus, and cerebellum as seen in representative sagittal sections Scale bar, 1 mm. Higher-magnification views of tdT+ cells in the boxed regions are shown in a1, a2, and a3. Scale bar, 200 μm. b Representative images of coronal sections showing the tdT-expressing cells in the ARH-A, ARH-C, and ARH-P in the tuberal region of the hypothalamus. Scale bar, 200 μm. [file 13041_2020_647_MOESM1_ESM.pdf]

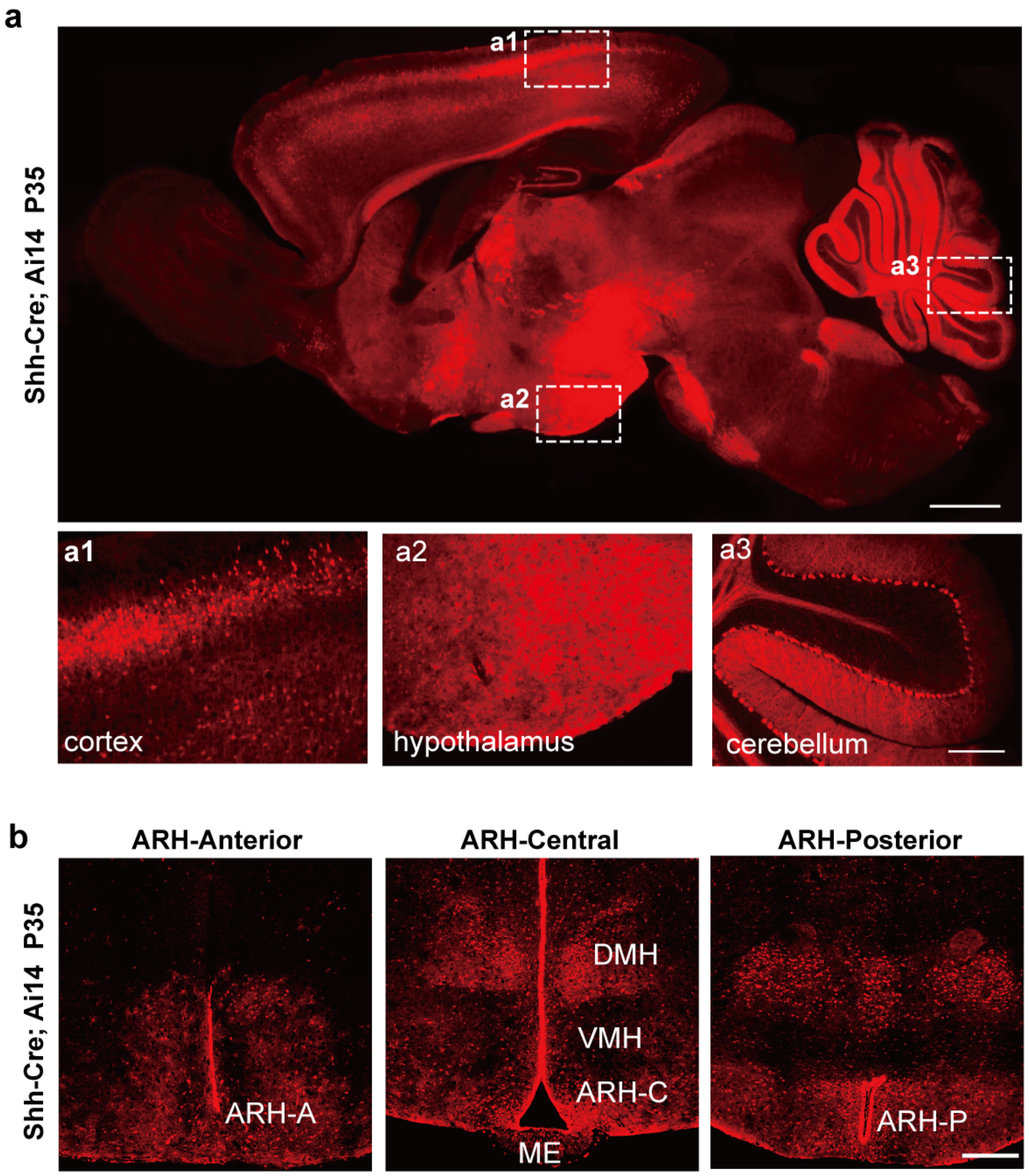

Fig. S1 Genetic tracing of the Shh promoter driving tdTomato (tdT) reporter expression in the brain of Shh-Cre;Ai14 mice at P35. **a** tdT-expressing cells are widely distributed in the cortex, hypothalamus, and cerebellum as seen in representative sagittal sections Scale bar, 1 mm. Higher-magnification views of tdT+ cells in the boxed regions are shown in a1, a2, and a3. Scale bar, 200 μm. **b** Representative images of coronal sections showing the tdT-expressing cells in the ARH-A, ARH-C, and ARH-P in the tuberal region of the hypothalamus. Scale bar, 200 μm.
