# Supplementary material for: Conditional knockout of leptin receptor in neural stem cells leads to obesity in mice and affects neuronal differentiation in the hypothalamus early after birth
Source: Mol Brain. 2020 Aug 3;13:109. doi: 10.1186/s13041-020-00647-9 (PMC7398062; doi:10.1186/s13041-020-00647-9)
Supplement: Supplementary file 2 — Additional file 2: Figure S2. Endogenous expression of pSTAT3 in Nes-cKO mice. Mice at P35 received 0.9% saline and were sacrificed after 1 h. pSTAT3 immunostaining of coronal brain sections showing pSTAT3+ cells in the ARH and ME in the hypothalamus. Scale bars, 100 μm. [file 13041_2020_647_MOESM2_ESM.pdf]

Ren et al., Figure S2

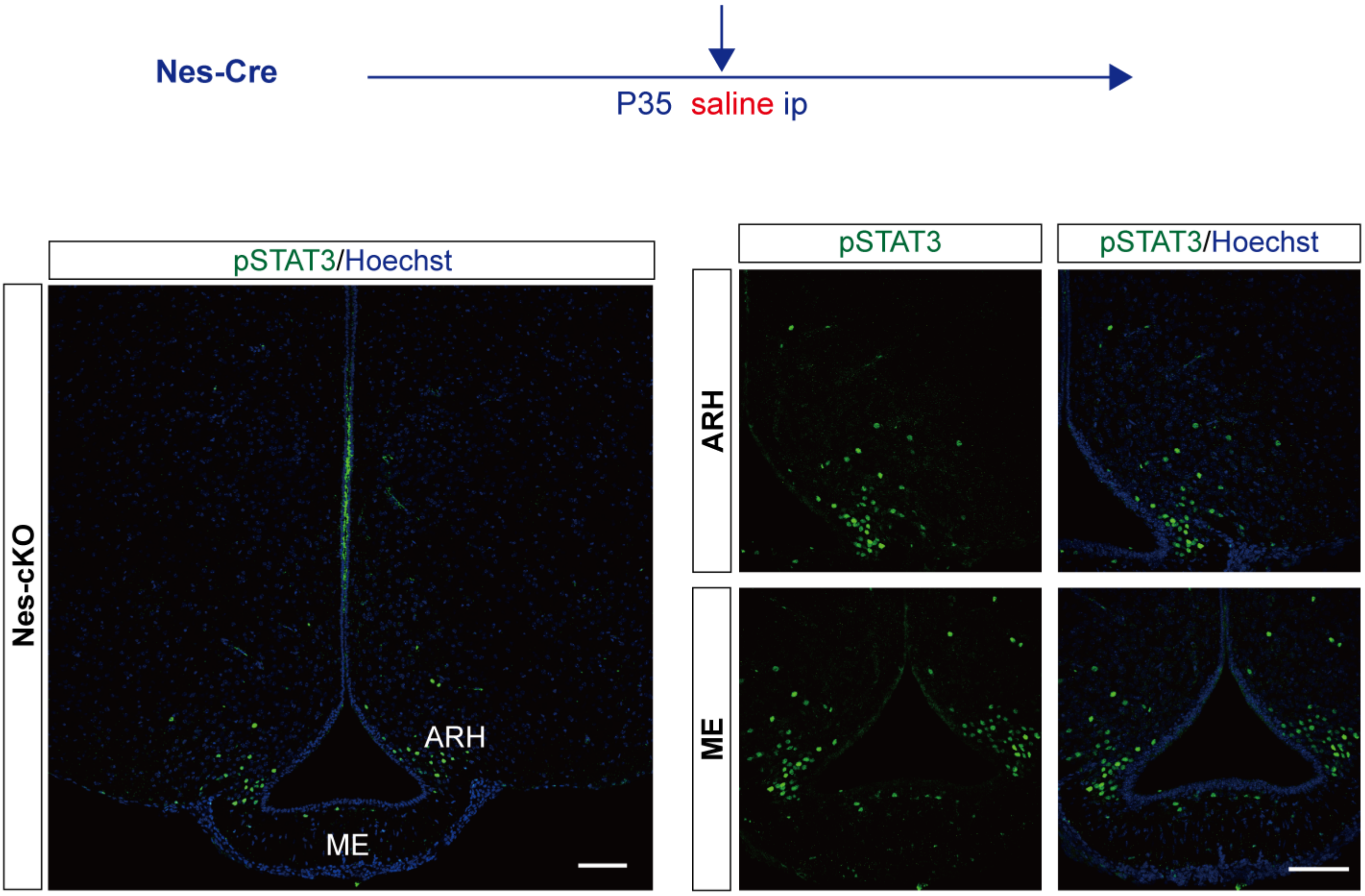

Fig. S2 Endogenous expression of pSTAT3 in Nes-cKO mice. Mice at P35 received 0.9% saline and were sacrificed after 1 h. pSTAT3 immunostaining of coronal brain sections showing pSTAT3+ cells in the ARH and ME in the hypothalamus. Scale bars, 100 μm.
