# Supplementary material for: Conditional knockout of leptin receptor in neural stem cells leads to obesity in mice and affects neuronal differentiation in the hypothalamus early after birth
Source: Mol Brain. 2020 Aug 3;13:109. doi: 10.1186/s13041-020-00647-9 (PMC7398062; doi:10.1186/s13041-020-00647-9)
Supplement: Supplementary file 3 — Additional file 3: Figure S3. Proliferation of cells in the ARH of adult mice. C57BL/6 wild-type mice at P60 were injected with BrdU twice daily for five consecutive days and then sacrificed. BrdU immunostaining showing the BrdU+ cells in the ARH-A, ARH-C and ARH-P. Scale bar, 100 μm. [file 13041_2020_647_MOESM3_ESM.pdf]

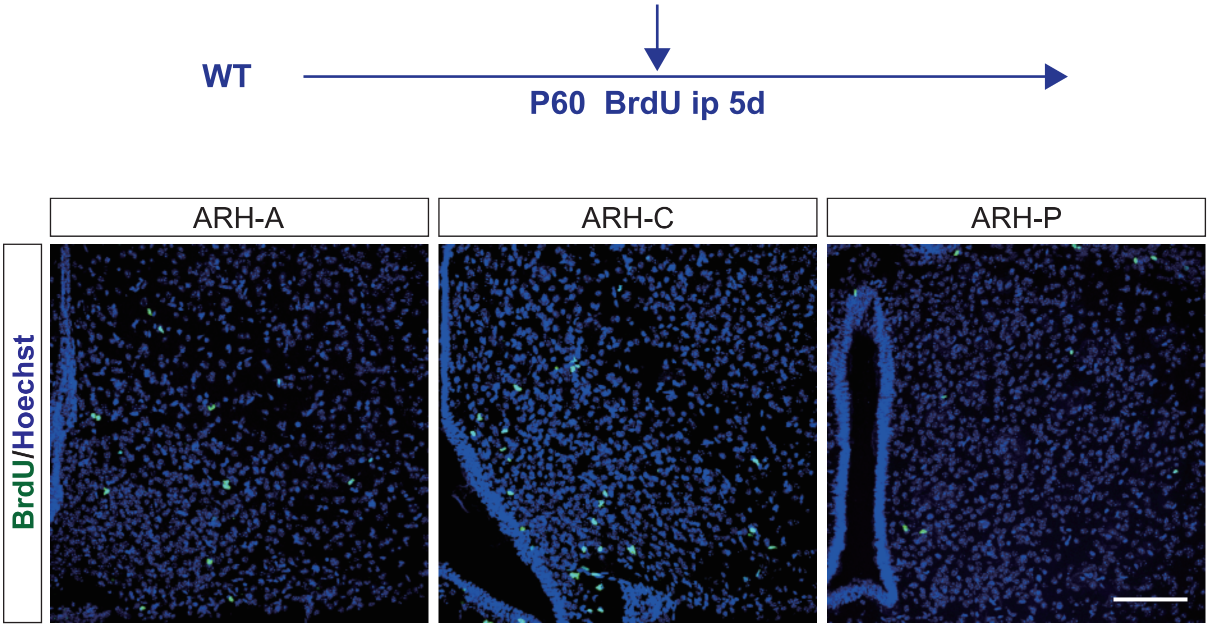

Fig. S3 Proliferation of cells in the ARH of adult mice. C57BL/6 wild-type mice at P60 were injected with BrdU twice daily for five consecutive days and then sacrificed. BrdU immunostaining showing the BrdU+ cells in the ARH-A, ARH-C and ARH-P. Scale bar, 100 μm.
